# Cognitive and Affective Assessment of Navigation and Mobility Tasks for the Visually Impaired via Electroencephalography and Behavioral Signals

**DOI:** 10.3390/s20205821

**Published:** 2020-10-15

**Authors:** Robert-Gabriel Lupu, Oana Mitruț, Andrei Stan, Florina Ungureanu, Kyriaki Kalimeri, Alin Moldoveanu

**Affiliations:** 1 Faculty of Automatic Control and Computer Engineering, Gheorghe Asachi Technical University of Iași, 700050 Iași, Romania; robert-gabriel.lupu@academic.tuiasi.ro (R.-G.L.); andrei.stan@academic.tuiasi.ro (A.S.); fungurea@tuiasi.ro (F.U.); 2 Department of Computers, Faculty of Automatic Control and Computers, University Politehnica of Bucharest, 060042 Bucharest, Romania; alin.moldoveanu@cs.pub.ro; 3 Institute of Scientific Interchange, Via Chisola 5, 10126 Torino, Italy; kyriaki.kalimeri@isi.it

**Keywords:** sensory substitution, cognitive load, brain activity, navigation, multimodal, audio, haptic

## Abstract

This paper presented the assessment of cognitive load (as an effective real-time index of task difficulty) and the level of brain activation during an experiment in which eight visually impaired subjects performed two types of tasks while using the white cane and the Sound of Vision assistive device with three types of sensory input—audio, haptic, and multimodal (audio and haptic simultaneously). The first task was to identify object properties and the second to navigate and avoid obstacles in both the virtual environment and real-world settings. The results showed that the haptic stimuli were less intuitive than the audio ones and that the navigation with the Sound of Vision device increased cognitive load and working memory. Visual cortex asymmetry was lower in the case of multimodal stimulation than in the case of separate stimulation (audio or haptic). There was no correlation between visual cortical activity and the number of collisions during navigation, regardless of the type of navigation or sensory input. The visual cortex was activated when using the device, but only for the late-blind users. For all the subjects, the navigation with the Sound of Vision device induced a low negative valence, in contrast with the white cane navigation.

## 1. Introduction

At the world level, approximately 2.2 billion people have a vision impairment or suffer from blindness, caused primarily by uncorrected refractive errors, cataracts, age-related macular degeneration, and glaucoma. The majority of people with vision impairments are over 50 years old, originating especially from low and middle-income countries [1]. The purpose of the Sound of Vision project (SoV) [2] was to develop an assistive system for the blind and visually impaired users that would facilitate navigation and obstacle detection. In this paper, we presented a study of cognitive load assessment and brain activation evaluation during an experiment in which eight visually impaired subjects performed various object detection and navigation activities while using both the white cane (a navigation aid they use on a daily basis) and the SoV device, which provided three types of sensory input—*audio cues* delivered through headphones, *haptic cues* delivered as vibrations applied on a vest that was placed on the user’s abdomen, and a combination of both audio and haptic information, called the *multimodal sensory input*. We performed a metrics analysis, cognitive load, working memory assessment, brain activity, visual cortex evaluation, and the identification of emotions during navigation in the real-world environment.

Section 2 presents an overview of mobility assistive devices, Section 3 introduces the biophysical signals and cognitive load, Section 4 describes the Sound of Vision device, Section 5 details the method, Section 6 presents the results and a discussion, and finally, Section 7 provides the final conclusions and future research directions.

## 2. Overview of Mobility Assistive Aids

The absence of visual information in the case of blind individuals can be substituted by conveying auditory and tactile stimuli, separately or simultaneously, through specialized assistive devices.

### 2.1. Auditory Vision Sensory Substitution

***Auditory vision sensory substitution*** (AVSS) devices [3] map the image “seen” by the camera into a matrix of active audio sources. The diversity of AVSSs is very large, ranging from optophone-like systems [4] to devices that use spatialized 3D sounds. The *optophone* (or the piano transform device) scans the image from left to right and converts the detected input into sound cues. The most well-known optophone is the vOICe [5], where the pixels’ vertical position is mapped to frequency, and their brightness is mapped to sound intensity. In other AVSSs [6,7], height is correlated to frequency distribution in the horizontal plane to binaural loudness, and brightness is encoded as sound intensity. In *pitch transform systems* [7,8], distance is related to sound frequency, while in *verbal transform systems* [7,9], objects are assigned to synthetic voice recordings. A problem of most optophone-like modern systems is that they overwhelm the users with too much output information, increasing cognitive load, effort, and concentration. This drawback can be overcome by reducing scene complexity, by maintaining only the salient characteristics and relevant objects, and by applying an effective sonification technique in order to provide the users a natural, effective, and easy to understand environmental representation. Modern AVSSs use binaural 3D sounds spatialized with generic (prerecorded, stored in large databases) or individualized head-related transfer functions (HRTFs). Individualized HRTFs are preferable for creating 3D sound as they are more accurate and fit the user’s auditory characteristics.

### 2.2. Tactile Visual Sensory Substitution

***Tactile visual sensory substitution*** (TVSS) systems use a matrix of controllable elements that provide spatial and temporal environmental information on the skin, either through kinesthetic or cutaneous sensations. In this type of device, a camera is used to acquire visual input that is consequently transformed into a tactile rendering via the multi-dimensional pin array, facilitating reading, shape, and face recognition [10,11,12]. One advantage of using TVSS devices is that the tactile sense, contrary to the auditory one, is less used and demanded in everyday activities. Thus, the user can receive cutaneous (awareness and stimulation of the outer surface of the body [13]) and kinesthetic (awareness of the limb position and displacement [10]) cues, without hampering locomotion or auditory perception at all [14]. On the other hand, a drawback lies in the fact that the capacity of the tactile channel is restrained to a limited maximum number of actuators and patterns to be applied. In addition, such devices cannot be used to a large extent because they are tiring and uncomfortable.

### 2.3. Auditory Tactile Visual Substitution Devices

When the scene is too difficult to be mapped onto the tactile array, the auditory channel is additionally recruited in order to enhance environmental representation, creating ***auditory tactile visual substitution*** (ATVS) devices. The first multi-sensory device was Nomad [15]. Tactile cues are delivered through a touch-sensitive tablet, and the auditory information consists of synthesized voice recordings. The Heard and Felt Vision Effects (HiFiVE) [16,17] system uses moving speech-like sounds (tracers—area tracers and shape tracers), binaural panning, and tactile effects in order to map visual images to an audio-tactile representation.

## 3. Biophysical Signals and Cognitive Load

### 3.1. Electroencephalography

***Electroencephalography*** (EEG) can provide neurophysiological markers of cognitive-emotional processes induced by stress and indicated by changes in brain rhythmic activity [18,19]. EEG signal processing techniques play a significant role in quantifying cognitive load [20,21,22,23,24]. Bos et al. [20] showed that cognitive load was an indicator of the learning progress. Berka et al. [22] extracted features from EEG signals for monitoring cognitive workload and task engagement. Nilsson et al. [23] showed learning outcomes from the subjects when they navigated a hypermedia environment. Scott et al. [24] also showed that a navigational map could create significantly more germane or extraneous cognitive load. Therefore, EEG/ Electrodermal Activity (EDA) signals are used to measure cognitive load and affective responses, and the overall process is explained in the following section.

*Cognitive load* and affective responses may impact the learning progress. The detection of reliable cognitive load and affective responses would improve the design of emotional intelligent mobility systems for the visually impaired people (VIPs). The complexity of the tasks is quantified in terms of cognitive load index and affective index, considering two well-established metrics in the scientific literature—the *event-related desynchronization (ERD)/event-related synchronization (ERS) index* and the *left-right asymmetry index*.

*Affective responses* directly influence the processes of cognitive learning. However, the challenges of learning can evoke negative affective responses [25]. Emotion assessment is a challenging and demanding task because people are not always able to express their emotions verbally [26]. Bos [20] showed that cognitive load could indicate changes in the learning process. He proposed an approach to determine the optimal placement of a limited number of electrodes, and then these electrodes were placed in an experiment aimed at determining arousal and valence. Left frontal inactivation is an indicator of a withdrawal response, which is often linked to negative emotion. On the other hand, right frontal inactivation is a sign of an approach response or positive emotion. High alpha activity (8–12 Hz in the EEG frequency band) is known to be an indicator of low brain activity.

Researchers have addressed the intertwining role of affective responses, learning, and cognitive load. Bower et al. [27] introduced the following hypothesis to study learning patterns: (1) a positive emotion usually increases the learning process through attention and motivation, (2) a positive emotion improves learning by enhancing cognitive load, and (3) a negative emotion decreases the learning process. Cattaneo et al. [28] employed the cognitive load theory for the understanding of the perceptual and neurocognitive mechanisms; however, there are still many open questions on how emotion and cognitive load can ease the learning process of the visually impaired people.

### 3.2. Electrodermal Activity and Heart Rate

Electrodermal activity (EDA) is a well-known indicator of physiological arousal and stress activation in affective computing [29,30]. It is more sensitive to emotion-related arousal variations as opposed to physical stressors, which can be better reflected by heart rate (HR) measurements. Blood volume pulse (BVP) patterns can also reflect transient arousal and cognition processes [31]. Two outdoor mobility studies from the early 1970s have suggested that some form of psychological rather than physical stress is responsible for visually impaired people’s increased HR versus sighted pedestrians [32,33]. However, certain mobility tasks (for example, stairs climbing) may result in an interactive psychological stress effect and momentary physical workload; thus, cardiovascular measures may be less suitable than EDA.

## 4. The Sound of Vision Device

Spatial navigation is a category of spatial cognition related to performing tasks, such as following paths, detecting obstacles, and reaching targets. It is based on developing, maintaining, and recalling an internal representation of the environment [34,35]. This internal representation depends on the spatial relation between entities and on the subject’s position, being classified into two categories: egocentric—the navigator is in the center of the coordinate system, and allocentric—the reference external to the navigator.

### 4.1. Technical Description

Sound of Vision is a wearable device that allows a visually impaired user to perceive and navigate the environment. It works by permanently scanning the environment, extracting essential features, and rendering them to the user through audio and haptic means.

The Sound of Vision final prototype includes an integrated custom hardware solution and a complex software solution, supporting the real-time operation of the device, as well as training tools and materials.

The hardware components of the system are:

− a headgear, including a 3D acquisition unit (depth camera for indoor or low light outdoor conditions, stereo camera for outdoor or bright light conditions, head and body inertial measurement unit (IMU) for body orientation) and an audio rendering unit (mounted on the head);
− a haptic belt with a matrix of 60 vibrating motors (six rows and 10 columns, placed on the abdomen);
− a processing unit: a small laptop with powerful CPU and GPU units (in a backpack);
− a wireless remote control (in the pocket).

When scanning the environment, the user can select from two different models for both audio and haptic: the *discrete model* (which renders the objects sequentially) and the *continuous model* (which provides real-time information at once about all the objects in the field of view). They are divided into sub-models and have different variations, as well as additional features for safe and reliable navigation: *Danger mode*—alerts about proximity objects and prevents collisions, *Flashlight*—enables the rendering of an object’s distance in front of the user, *texts and special signs detection* and *best free space*—which indicates a navigable opening between surrounding objects.

The user movement is guided solely by the Sensory Substitution Device (SSD) with no additional feedback from the assistant or from other sources (i.e., maps from Google or GPS coordinates from a GPS device). The SSD device generates audio and haptic signals that are an encoded representation of the environment in the proximity of the user. Through intensive training, the user gains fluency in understanding the audio and haptic encoded feedback issued by the SSD device, and then he/she can make proper decisions for further movement in the environment.

Like any other person, the VIP wants to walk in the direction of the sound source. The SoV device scans the environment, detecting the obstacles and their features, and sends audio or/and haptic stimuli to users. These stimuli help the VIP to avoid obstacles and to find a secure path to the sound source. Obviously, the real scene and the stimulation are changed/updated according to the VIP’s route, like in a maze. Depending on their perception, the VIPs may choose different paths.

### 4.2. The Focus of the Study

In line with the SoV project overall goals, the aim of this study was to evaluate the VIPs’ cognitive load and emotional stress in real-world mobility experiments, in two cases: navigation with white cane and navigation relying on the SoV prototype with audio, haptic, or audio and haptic (multimodal) codification. The research questions we pursued were based on the following comparative assessments of cognitive load:

- when using the Sound of Vision device with audio vs. haptic vs. multimodal input;
- when using the Sound of Vision device vs. white cane during a navigation task in the real-world environment.

Based on some achievements presented in scientific papers and on the valuable previous experience [36,37,38,39], the experiments were oriented to collect EEG and physiological (EDA and HR) signals in five different mobility tasks in order to highlight the VIPs’ cognitive load and stress in correlation with some events (collisions or total confusion) captured from the recorded videos.

As presented above, it has been proven by many studies that EEG is a promising and common approach to measuring cognitive load (CL), working memory load, emotional states, and any cerebral signals denoting cortex responses to specific stimuli. CL was an effective real-time index of task complexity backed up by behavioral evidence. Complementary, the peripherical physiological measurements reflected arousal and stress activation (EDA) and transient processes in arousal and cognition (HR). The mobility tasks did not include a consistent physical effort, so HR could be also considered in a multimodal approach.

An important aspect of this work was related to the VIPs’ preference and long-term accommodation to navigating using the white cane, as they were educated to use it from the moment they lost sight or from childhood. Although the SoV device offered much more information about the environment (the number of nearby objects, their position and properties, the presence of specific objects, and so on), it was expected that for the first tests, the VIPs would have lower cognitive load and better performances during the benchmark task with the white cane than with audio or haptic stimuli. Obviously, the length of the accommodation period with the SoV device depended on each VIP’s education and ability to learn. It was important for this study to understand how easily the SoV stimuli were perceived and processed by a VIP and which navigation modality (audio, haptic, or audio and haptic) was less stressful and more quickly accepted. We expected that audio mobility would outperform the haptic and fusion mobilities, knowing that blind people generally have a well-developed hearing sense.

Additionally, the VIPs’ visual cortex excitation by audio and haptic stimuli during navigation was investigated, expanding the existing literature [40,41] that has reported brain activity in the visual cortex during EEG measurements for blind people who have received visual information through sensory substitution devices (SSDs).

To our best knowledge so far, this paper was the first one to present a comparative study regarding VIPs’ stress, cognitive load, and visual cortex excitation while navigating in the real-world using a common white cane vs. a sensory substitution device.

## 5. Materials and Methods

This experiment has been carried out using the Acticap EEG device with 16 electrodes, provided by Brain Products GmbH from Germany and Shimmers Multisensory provided by the Shimmer Sensing company from Dublin, Ireland.

### 5.1. Experimental Setup

**Figure 1:**
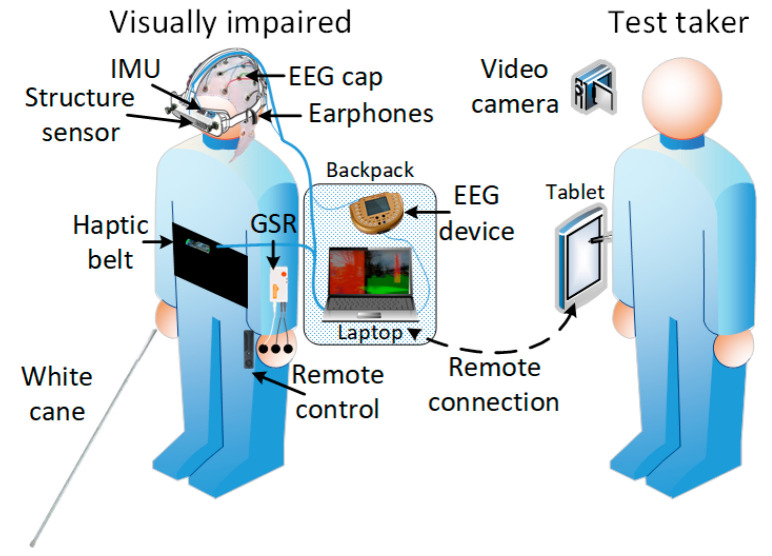
Real-world setup. IMU- Inertial Motion Unit, EEG–Electroencephalography, GSR–Galvanic Skin Response.

The aim of the experiment was to obtain a dataset, as large as possible, with EEG and physiological signals during the trials designed for traveling in fixed scenarios with the help of the SoV device [42]. There were two user setups. The first one was the **virtual training environment (VTE)** setup that was used to train the subjects and accommodate them with the audio and haptic encoding models prior to using the system in real-life scenarios [43,44]. The second setup (Figure 1) was the **real-world (RW)** setup used in an indoor controlled environment and outdoors.

The difference between the VTE and RW setups consisted mainly of the video streaming sources that feed the processing and control unit. For VTE, the video stream was provided by a virtual reality serious game in which the VIP navigated using the keyboard or a joystick. In the VTE tests, the VIP wore the headset consisting of a structure sensor stereo video camera and an IMU sensor [45,46], but only the IMU signal was used in order to orient in the virtual environment by head movements. For the RW setup, the video stream was provided by the structure sensor (indoor use of the system) or stereo video camera (outdoor use of the system).

**Figure 2:**
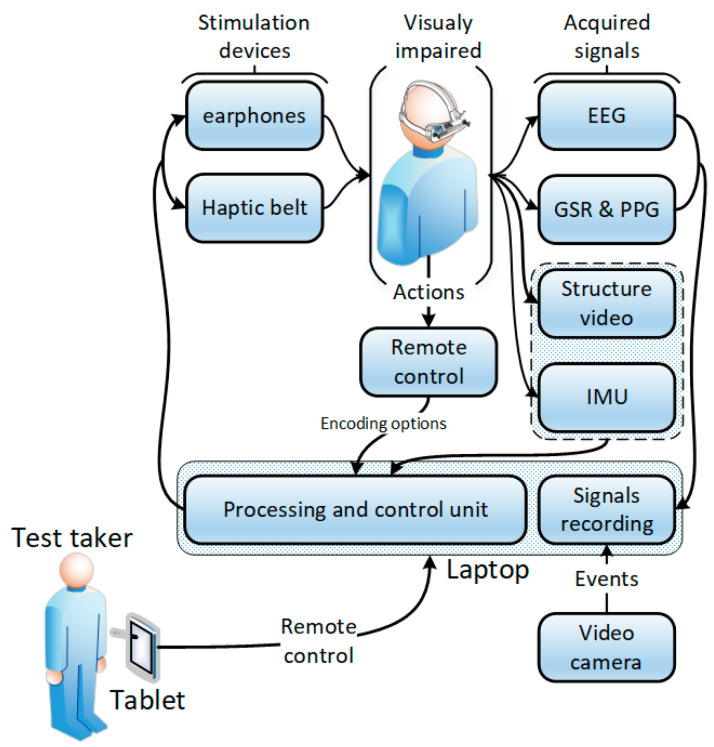
The system architecture for the real-world (RW) setup. PPG-plethysmography.

The SoV system used in the RW experiments is depicted in Figure 2 and consisted of two computing systems: the first one was the processing and control unit (PCU) attached to the VIP, and the second one (the tablet from Figure 2) was used by the assistant who controlled how the trial was performed. Via a remote connection with the PCU, the assistant could adjust parameters, select scenarios, and enable physiological signals recording. The PCU ran the SoV runtime application, which sensed the environment and provided audio and haptic stimuli. It also recorded physiological data from the user who performed the navigation tasks. The IMU signals were used to determine the user’s body and head orientation that was further used to render the audio and haptic output in accordance with the RW scene. The VIP had the opportunity to select the most appropriate audio or haptic encoding by using a remote control connected to the PCU. During navigation, there was no communication between the VIP and the assistant. The VIP walked autonomously based only on the stimulation provided by the SSD. The test taker’s task during tests was to ensure that the system was working and that the VIP received correct clues. He did this by using a tablet connected wirelessly to the VIP’s laptop.

We used a lightweight laptop (Dell XPS) and two cooling fans in order to cool down the laptop, which are part of the SoV prototype for experimental purposes. The experimental system (laptop, battery, fans, and EEG device) weighed no more than 3.5 kg, and the users did not feel uncomfortable due to the weight and/or heat. Actually, a lighter version of the SoV system—more energy-efficient and low cost—is under development.

**Figure 3:**
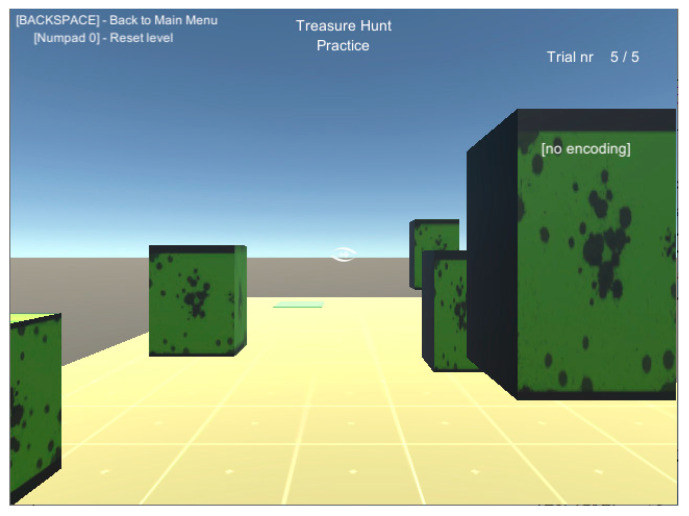
Treasure hunt in a virtual training environment (VTE).

**Figure 4:**
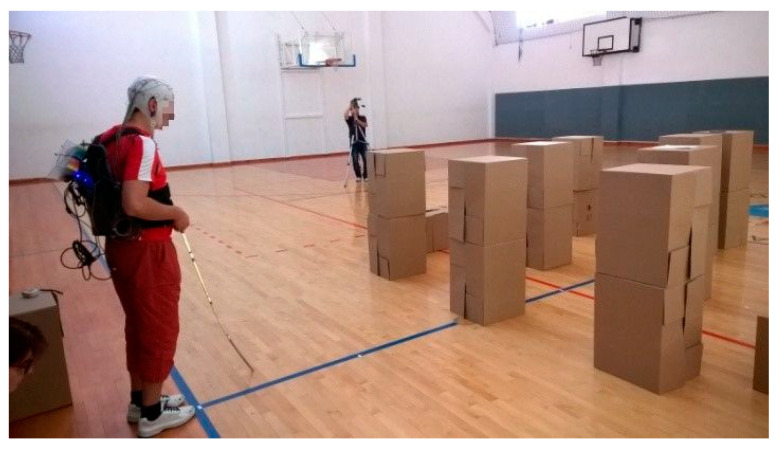
Treasure hunt in RW.

A print screen from the serious game called **“treasure hunt” (TH)** is presented in Figure 3. The VIP had to use the joystick in order to position himself in the virtual scene, exactly where the sound source originates. In RW, the user had to navigate through an indoor environment in order to reach a target sound source while avoiding cardboard box obstacles of various dimensions (Figure 4).

We conducted EEG and EDA/HR recordings during experimentation in RW, under two conditions: **white cane only** (for the participants who used this mobility aid on a regular basis) and **SoV device only**. The scenes were tested using the *audio encoding, the haptic encoding, and both the audio and haptic encodings (multimodal)* with the SoV device, 5 trials each. In order to minimize the required testing resources, the users had the opportunity to choose the sonification model and tactile stimulation [47,48,49] that best suited their level of perception and understanding.

The **static scenes (1R)** were tested with the *discrete model*, while the **dynamic scenes (TH)** were tested with the continuous *model* in both virtual and real-world environments.

In the *discrete (or iterative) model*, the scene was rendered in a loop, one by one. A sphere was constantly expanding its radius until 5.25 m with a speed of 2 m/sec. Auditory and tactile stimuli were provided when this sphere intersected scene objects, allowing distance detection, as well as comparing the distance between objects. The continuous model rendered an entire scene at once, providing instantaneous information via audio and haptic.

VTE tests were recorded automatically by the SoV system, while RW tests were recorded manually by testing assistants. Furthermore, each trial in a test was videotaped for annotation purposes. The time needed to finish every trial and the accuracy were saved: number of collisions between user and obstacles, number of cane contacts with objects, time duration, together with path length followed.

**Figure 5:**
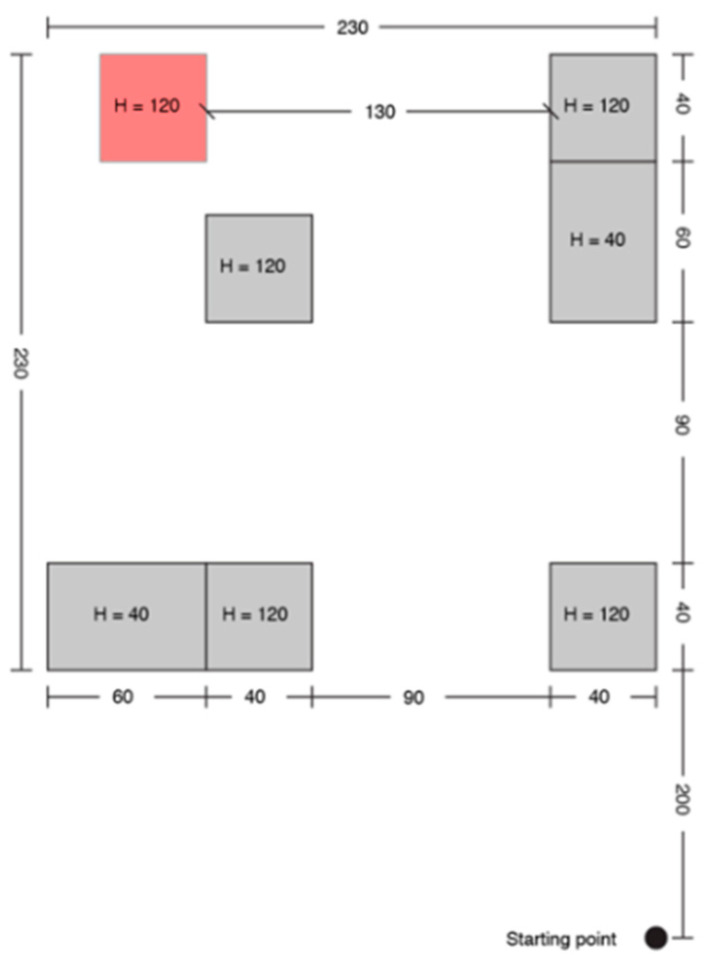
Treasure hunt (TH) configuration A.

**Figure 6:**
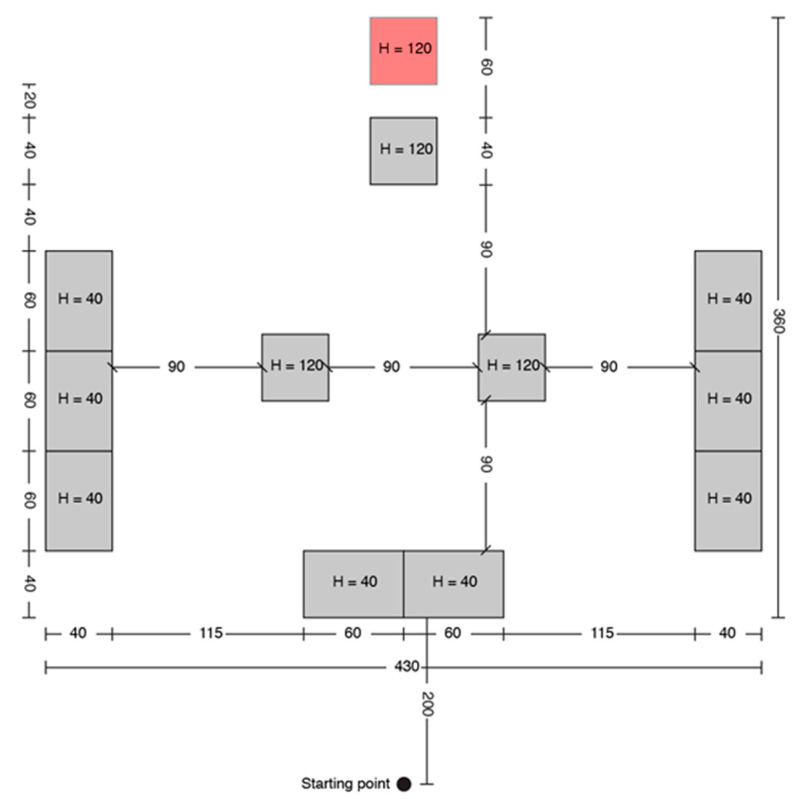
TH configuration B.

**Figure 7:**
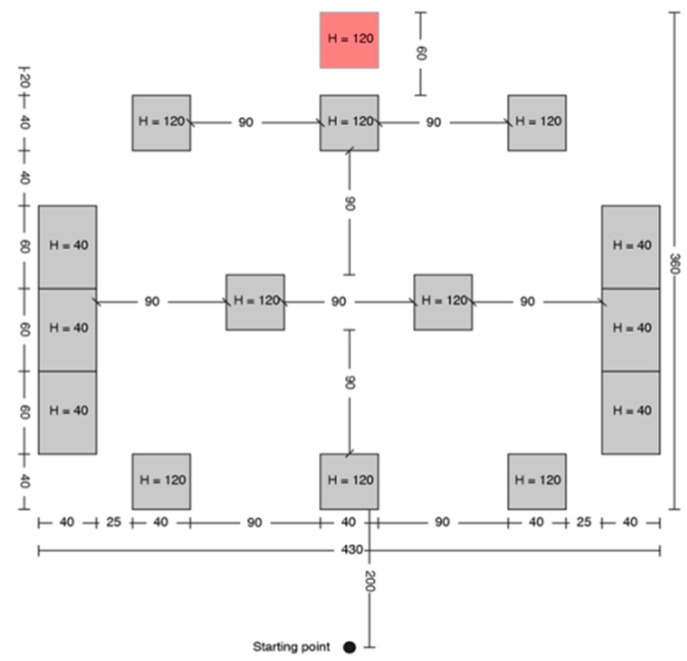
Treasure hunt configuration C.

**Figure 8:**
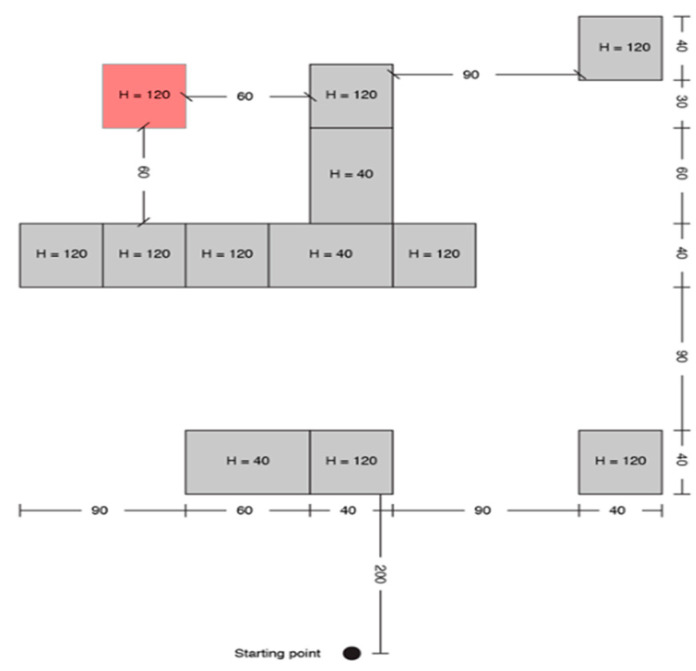
Treasure hunt configuration D.

**Figure 9:**
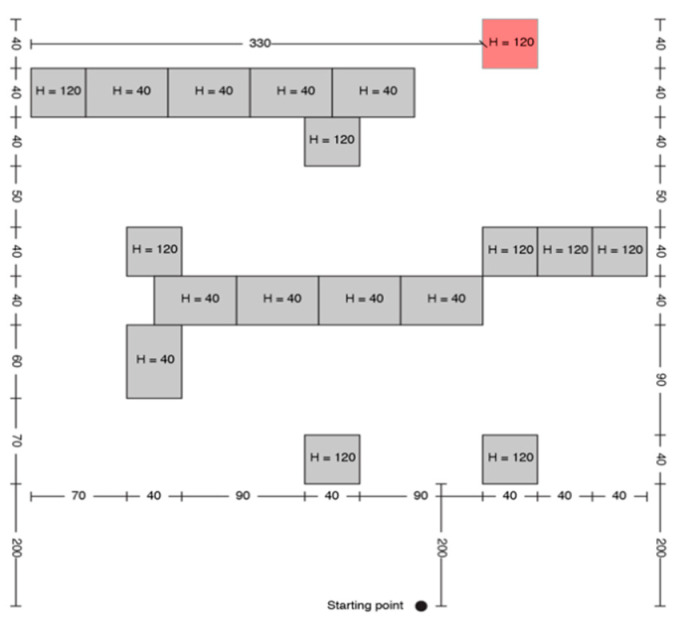
Treasure hunt configuration E.

Each test consisted of 5 trials, and each one was assigned to a fixed path/boxes arrangement for TH, as it is presented in Figure 5, Figure 6, Figure 7, Figure 8 and Figure 9.

### 5.2. Data Collection

In this study, 15 VIPs were involved in training and testing tasks in the virtual environment and real-world settings, using the SoV prototype and different releases of the SoV runtime. After each testing stage, important improvements were made to the hardware and software resources, including audio and haptic encodings based on the VIPs’ feedback. Finally, only 8 complete datasets (corresponding to 3 females and 5 males, aged 20–42) with fully validated data were retained and subsequently analyzed. All the participants provided informed consent approved by the research ethics committee of the institutions involved in the project (Approval number: 9083/15.05.2017). One hour before performing the tests, the VIPs did not drink coffee nor black tea, and also smoking was forbidden before or during the experiments.

For EEG and additional physiological measurements, the following equipment was used:

- a BrainProducts V-Amp 16 amplifier and an EasyCap helmet with 19 sintered Ag/AgCl miniaturized passive electrodes for EEG signal acquisition with a sampling rate of 512 Hz;
- a Shimmer3 GSR+ unit sensor for measuring electrodermal activity/galvanic skin response (EDA/GSR) and continuous HR;
- a video camera or smartphone for video recording in real-time.

The acquisition procedure used 16 electrodes, namely Fp1, F7, F3, C3, P3, P7, C4, O1, O2, Fp2, F8, F4, C4, P4, T8, P8, and an ear reference, placed according to the 10-20 international system. The sampling rate was 512 Hz, and the AFz electrode was connected to the ground. To ensure reliable EEG raw data, the impedance of each electrode was maintained below 5 kΩ, by using a good abrasive gel. The OpenVibe open source software was used for EEG acquisition. The OpenVibe server acquired the EEG signals, and the OpenVibe client saved or sent the raw data as a stream. The Shimmer GSR unit sent the acquired data via Bluetooth.

**Figure 10:**
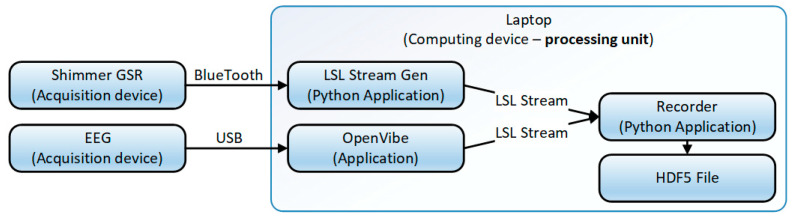
The data acquisition process. LSL, lab streaming layer.

The data acquisition process is outlined in Figure 10. An important part of the acquisition process is the usage of the lab streaming layer (LSL) protocol [50] so that each data component had to provide a stream of data as output. For the components that do not natively provide LSL output streams, simple adaptors had to be designed, as in the case of the data provided by the EDA/GSR device. The EEG data was available as an LSL stream provided by the OpenVibe application. The applications developed in this project (VTE and SoV runtime) provided LSL streams to source events that were internally generated and of interest for later analysis. The data was stored in a tabular format inside hdf5 files [51], together with a timestamp provided by the LSL.

In what concerns video recording and annotations, the mp4 video files recorded during the tests were annotated with Chronoviz [52]. The events: *Start* (beginning of the recording), *Collision*, *Find* (find the source/treasure), Lost (lost control), *TouchCane* (only for the test with the white cane), Stop (end of the recording) were considered. For each mp4 file, a CSV file with annotated events and corresponding Unix timestamps was generated.

**Figure 11:**
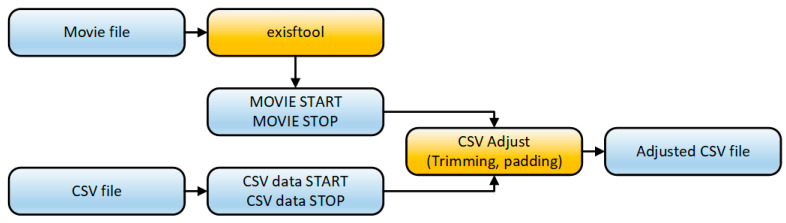
RW events synchronization process.

Alternatively, if Chronoviz could not be used due to system constraints (Chronoviz needs a system with Mac OS X 10.6 or later), a Python script was designed to synchronize the data streams acquired by the processing unit with the video recordings of the experiment. The script aligned the timestamps of the samples in the acquired data streams (provided as csv files) with the timing information found in the video recording. The application ExifTool [53] was used for gathering timing information from the movie files. The data streams were trimmed or padded in order to fit the movie length. The script detected and reported any timing misalignments and provided means to fine-tune the synchronization process. The resulting adjusted data streams and movies could be annotated later in a similar way as in Chronoviz. The synchronization process is presented in Figure 11.

### 5.3. Data Acquisition and Preprocessing

The acquired brain waves were pre-processed. We applied a band-pass filter for 0.5–100 Hz, a notch filter to remove power line contamination at 50 Hz, and a band-pass filter to obtain frequency bands of interest (delta, theta, alpha, beta, and gamma). The artifacts (involuntary eye blinks, muscle movements, brief amplifier saturations) presented in the EEG signals were removed using an online Savitzky-Golay filter. The EEG data obtained after pre-processing were baseline-normalized by subtracting for each participant and for each channel the mean of the resting state recordings (recorded in the laboratory during the VTE sessions).

The Shimmer software for EDA acquisition could not be efficiently used in the SoV setup (Figure 1), and therefore the streams from the sensor were acquired over Bluetooth at 16 Hz. The skin resistance values (y, μS) were computed from the Shimmer ADC values with the following linear function:

(1) y=p1×x+p2

where p1 and p2 are parameters specific to the range setting and can be selected from the datasheet of the sensor. If the electrodes are not tightly attached and lose contact with the skin, motion artifacts (high-frequency noise) can be present in the acquired signals. A low pass filter was applied to remove high-frequency noise, which can be attributed to movement artifact and other noise components. A cutoff frequency of as low as 1–5 Hz could be used without affecting the data of interest due to the slowly varying nature of the EDA responses.

### 5.4. Data Analysis

Within the broader framework of the SoV project, the aim of this study was to explore the VIPs’ brain activity during navigation tasks with the help of an SSD based on audio, haptic, and multimodal encoding, compared to white cane navigation. The research was focused on assessing cognitive load, visual cortex excitation, and emotions evaluation during RW navigation. For each exploration, the EEG signals were selected according to the analyzed brain lobes and the *power spectrum, and the asymmetry between the two cortex hemispheres was calculated.*

Usually, CL is investigated in the channels corresponding to the frontal lobe, which reflect the activity of short-term memory and consists of calculating **frontal-asymmetry**, meaning the difference between the logarithms of the power spectrum of the left and right hemispheres divided by the logarithm of the total power spectrum of both hemispheres. There is no single standard way to calculate asymmetry, and some authors use the difference or the ratio between the spectral powers of the signals on the right and the correspondents in the left hemisphere. Anyhow, *higher asymmetry reflects a strong workload, while lower asymmetry reflects avoidance and relaxation* [54].

CL is strongly related to emotional well-being states. The “feeling good” aspect of well-being deals with the balance of positive emotions vs. negative emotions. Well-being reflects a person’s ability to identify and respond to the challenges of everyday life, even painful and unpleasant events [55]. Hawthorne presented an extensive study on how feeling good might contribute to cognitive load in different ways [55].

Certain states can be more accurately investigated if the EEG waves are analyzed in the five specific bands: *delta, theta, alpha, beta,* and *gamma*. The *delta* waves reflect the unconscious states, and it is usually recorded in deep dreamless sleep. The *theta* waves are typically associated with the subconscious mind, sleeping, dreaming, meditation, or even artistic creation. The *alpha* waves are visible in all the cortex lobes and give valuable information regarding brain activation and the relaxed (but yet aware) mental state. High alpha activity has been correlated to brain inactivation. The *beta* waves are correlated to high mental activity, more prominent in the frontal cortex but visible over other lobes as well. The alpha and beta waves are the most used to classify workload using EEG. The *gamma* waves (>30 Hz) reflect hyper brain activity and have become more and more studied as the sampling frequency of the acquisition systems has increased [56].

Regarding **visual cortex (VC) excitation**, it must be specified that it was not known before the year 2000 whether the visual cortex could receive input from other sensory modalities besides the eyes through the lateral geniculate nuclei. Afterward, the EEG measurements have revealed that the VC activity is higher for blind subjects during rest or auditory/tactile tasks than in normal control. Without a certain demonstration, Sadato et al. suggested that in blind subjects, the cortical areas normally reserved for vision might be activated by other sensory modalities [57]. In 2003, Burton reviewed various brain imaging studies, which investigated the visual cortex activity of VIPs during nonvisual tasks, such as hearing messages, Braille reading, or even sensory discriminations of tactile or auditory stimuli, and concluded that the loss of vision did not lead to a permanent inactivation of the visual cortex [58]. A scientific report from Georgetown University Medical Center concluded in 2010 that “people who have been blind from birth make use of the visual parts of their brain to refine their sensation of sound and touch” [59]. In recent years, several studies have highlighted enhanced auditory processing in blind persons to partially compensate their impairment, with greater sensitivity of the other senses. It has been proved that the VC plasticity allows this cortical lobe to be colonized by the auditory and somatosensory systems in the case of congenitally blind persons. The study conducted by Campus et al. revealed that the occipital activation to sound was strong in sighted persons and much lower in blind persons [60]. Another valuable conclusion was that the occipital lobe of sighted subjects played a major role in the reconstruction of the environmental spatial metrics and that vision loss blocked this process. Obviously, it is expected to remark differences in VC excitation between the people who are blind from birth and those who lost their sight later and know what color, distance, or shape mean. For this analysis, the O1 and O2 electrodes are the most important, but also the Oz and the electrodes from parietal lobes should be considered in an extensive study.

In terms of evaluating emotions, it is well known that the **amygdala** is responsible for the perception of emotions, such as anger, fear, and sadness. The pre-frontal cortex and the hippocampus (located in the medial region of the temporal lobe) are highly correlated to emotional activity [56,61]. Because the right hemisphere is associated with negative emotions (i.e., fear or disgust), and the left hemisphere is highly activated by positive emotions and motivation (i.e., happiness and satisfaction), the EEG asymmetries in the frontal and parietal lobes are relevant for valence and arousal assessment [56]. A thorough evaluation can be performed if the signal analysis is performed on the EEG frequency bands of alpha, beta, and gamma. According to these findings and based on some other studies related to efficient EEG channels selection for emotion recognition, Zhang and his coworkers recommended the following set of electrodes: Fp1, Fp2, F7, F8, C3, FC5, FC2, AF4 (frontal lobe), T7, T8 (temporal lobe), O1, Oz (occipital lobe), and P3, P4, Pz, PO4 (parietal lobe) [62]. For emotions assessment in this study, only the channels C3, C4, T7, T8, P3, P4, F3, and F4 were considered due to the limited number of electrodes of the EasyCap helmet. The asymmetry in the pre-frontal lobe was presented in CL evaluation, and O1 was not considered because the standard list refers to sighted people, and, in our approach, the visual cortex was subjected to special attention.

## 6. Results and Discussion

### 6.1. Navigation Metrics Analysis

As mentioned above, the EEG, HR, and EDA (GSR) signals were acquired for the treasure hunt tests, using the white cane or the SoV device with three spatial information encodings—audio, haptic, and multimodal (audio and haptic). Besides the video recordings and the files containing the data obtained during the experiments, important metrics regarding navigation were collected for each user involved in the study: the *time required to accomplish a trial, the length of the path, the number of major or minor collisions,* and also *the numbers of white cane contacts with the obstacles*. All these data are summarized in Table 1 and reflect the cumulative performance of all the users for each scenario type.

**Figure 12:**
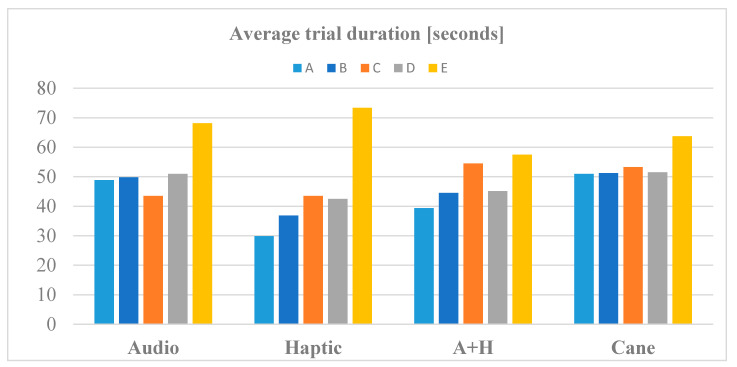
The average time to perform navigation tasks.

**Figure 13:**
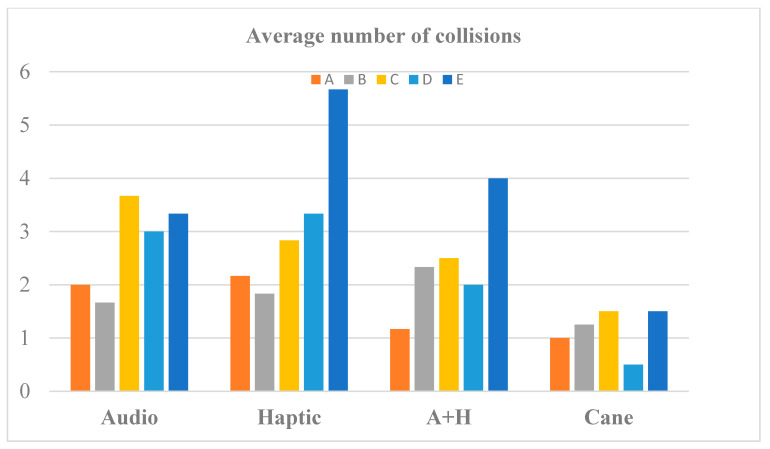
The average number of collisions during navigation tasks.

**Figure 14:**
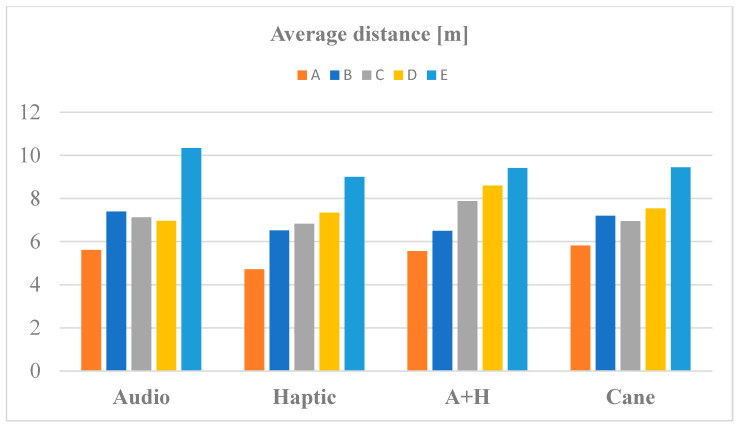
The average distances of navigation tasks.

Figure 12, Figure 13 and Figure 14 present the averages of time duration, number of collisions, and traveled distances for RW navigation with the help of the white cane and SoV device, in case of all the five obstacle arrangements (A to E). A+H stands for audio and haptic (multimodal). In the case of the short and easy routes, the walking durations were very similar for audio stimulation and cane traveling, while the haptic and multimodal stimulation required less time than the white cane. Only for the most complicated test scenario (E), the cane and multimodal tasks were performed in a shorter time than with haptic and audio input. It is known that the VIPs usually walk slowly, and it was encouraging that the SoV device did not slow down the movement of the users.

We noticed a higher number of collisions when using the SoV device in comparison to the white cane navigation. This fact was expected because usually, the VIPs touch the objects with the cane along their path, avoiding the great majority of collisions. The average distances did not differ too much between cane and SoV navigation, except for the SoV audio mode, for which the routes were significantly longer regardless of the testing scenario.

From Table 1 and Figure 12, Figure 13 and Figure 14, it can be concluded that the required time, the length of the path chosen by each VIP according to his/her perception of the SoV stimulation, and the number of collisions depended on the complexity of the scene and on the user’s training and ability to adapt to a new navigation aid. Obviously, the time, length of the path, and the number of collisions were much higher for the scenes C, D, and F. Some VIPs had better results with the audio mode and others with the haptic mode, but the number of collisions was higher for the haptic mode. As expected, the metrics for the white cane navigation were better because the VIPs were accustomed to using it on a daily basis. As a particular conclusion, *the VIPs’ navigation performance with the SoV device was better in the case of the multimodal encoding, in terms of duration and number of collisions*. On the other hand, no general conclusion could be drawn because the number of VIPs involved in the experiments was small, and also a VIP could have learned the scenes during the first trials and performed better during the last trial, even if the experiments were randomly conducted.

### 6.2. Cognitive Load Analysis

**Figure 15:**
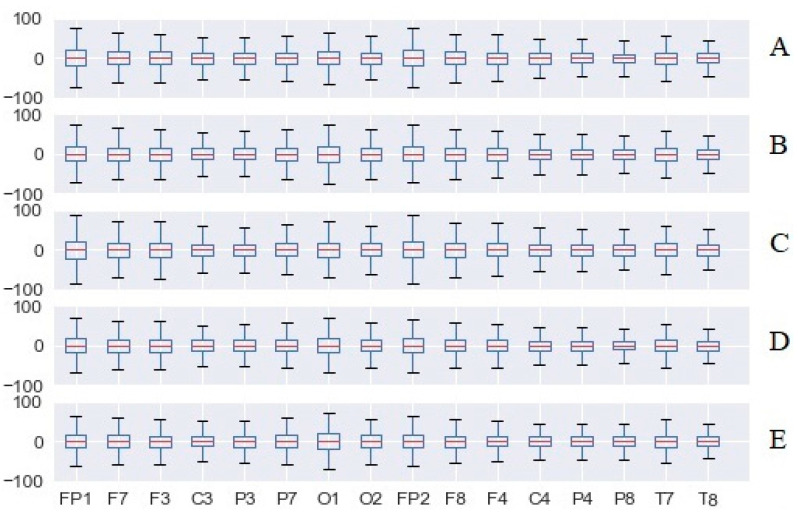
The total cognitive load of all electrodes for white cane navigation.

**Figure 16:**
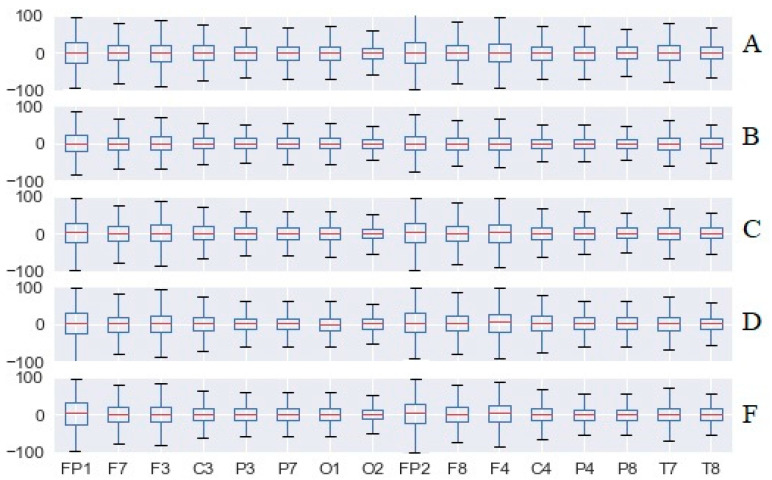
The total cognitive load of all electrodes for Sound of Vision (SoV) navigation.

Figure 15 and Figure 16 present the total cognitive load for all the validated experiments and the five test scenarios, in the case of both the SoV device and white cane navigation. In the case of SoV navigation, we computed the average of the audio, haptic, and multimodal stimulations. Regardless of the difficulty of the test scenario (A is the easiest, and E is the most difficult), high values of CL were observed for the electrodes related to the frontal cortex (O1 especially, in the vision area) if the SoV device was used. The increase of frontal cortical activity was expected, but the activity of the visual cortex (VC) was worth being investigated because it supports some previous opinions about VC activation in the case of the VIPs who received various environmental sensory stimulation. It should be noted that the brain activity corresponding to the O1 channel was significantly higher than for the O2 channel for both types of navigation, resulting in an increased emotional state.

**Figure 17:**
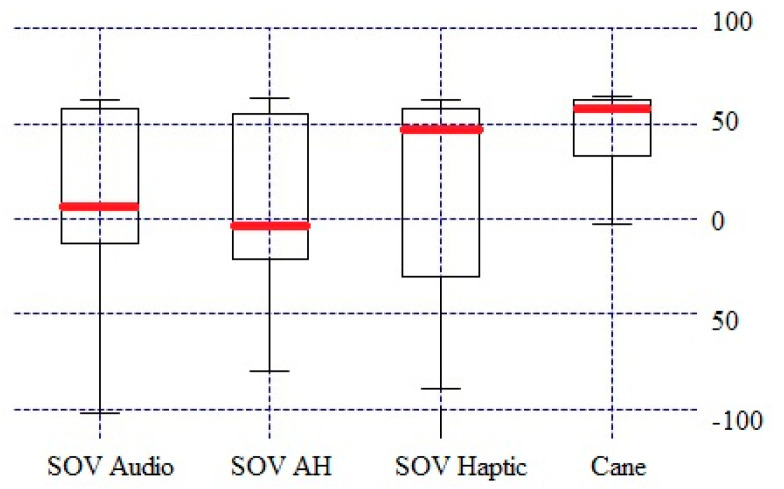
The total cognitive load (CL) for the TH—configuration C task.

In Figure 17, we present the total CL for the scenario treasure hunt (TH), configuration C. The main conclusion was that there was a *significant increase in the CL index* (indicated as a negative fluctuation according to the CL index definition) *in the case of using the SoV device with audio, haptic, and multimodal stimulation in comparison to white cane navigation*. The conclusion was similar in the case of the other testing scenarios (TH, configurations A, B, D, and E).

In contrast to the CL values presented in Figure 15 and Figure 16 for each electrode, the global CL index was calculated on average for all brain waves for all users, aiming to have a general representation of the brain activity. The short box related to cane traveling meant that the data consistently hovered around the center value, denoting a similar effort for all users, and the whiskers indicated a quite limited distribution as well. In the case of using the SoV device with audio, haptic, and multimodal stimulation, the taller boxes indicated more variable data, and the whiskers showed a wider distribution, namely more scattered data. The different ways in which the users perceived the haptic and sonification models could explain this conclusion, which anyway was in accordance with the plots depicted in Figure 12, Figure 13 and Figure 14. The consistency of the experimental data and the preprocessing accuracy were proved by the lack of outliers in the total CL indexes. Although the median value for haptic stimulation was closer to the median for cane walking, the distribution of global CL was the widest one. It could be observed that multimodal stimulation had the effect of reducing the spread of the global CL index. The tactile and auditory stimuli were processed by distinct lobes of the cerebral cortex with significant differences in CL, and this should explain the negative skewness in the case of cane and haptic stimulation and the positive skewness in the case of audio and multimodal stimulation. The conclusion was similar for the other testing configurations (A, B, D, and F) of the TH scenario.

### 6.3. Brain Activity Analysis

Besides this general evaluation of cognitive load, it was relevant to explore how the VIPs’ cortical lobes were activated during walking on certain routes with the white cane or guided by the SoV device using the three input encodings. Particular reactions were expected, depending on the type of visual impairment and on the users’ training or education. For this, the analysis of the individual frequency bands was performed according to the literature guidelines. First of all, *the alfa waves* were investigated, especially in the *frontal lobe*, taking into account that there is an *inverse relationship between alpha power and cortical activity;* namely, more brain activity (engagement) means less alpha power [63]. A more detailed analysis should be done if the alpha-1 (lower alpha, 7–10 Hz) and the alpha-2 (higher alpha, 10–13 Hz) frequencies were considered because it is well known that *alpha-1 is related to response inhibition and attentional demands,* and *alpha-2 reflects task performance in terms of speed, relevance, and difficulty* [64]. It has been proved that people with relatively *increased left-frontal alpha activity are more motivated and focused in a positive way,* and their related emotions are joy or anger. In contrast, *the increase of right-frontal activity denotes a more negative motivation accompanied by fear, sadness, and disgust* [63,65]. The asymmetry was calculated based on the difference between the logarithms of the spectral powers from the left and right brain hemispheres.

For a more accurate assessment of brain activity related to users with different perceptions and visual impairments, the envelopes of the alpha1 and alpha2 bands asymmetries were depicted for the considered navigation tasks, and the collisions annotated with Chronoviz were marked with black dots. It must be mentioned that the acquired signals for navigation with the cane or with the SoV device had different lengths, according to the time required to perform the task and the path chosen by the user, as it is presented in Table 1. The brain activity exploration was oriented towards analyzing the late visual impaired users in a group and the subjects who were born blind in another group.

**Figure 18:**
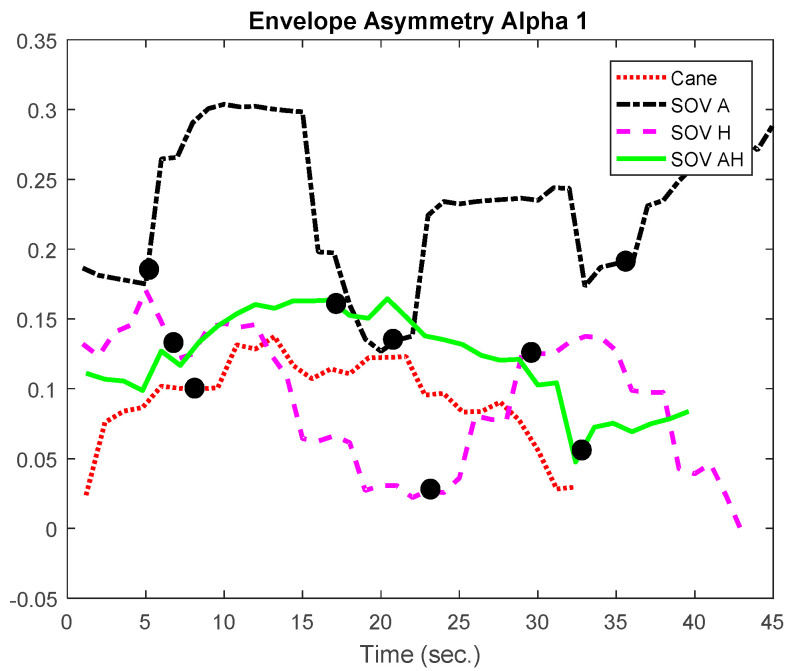
The envelopes of the alpha1 asymmetries for a user who was born blind.

**Figure 19:**
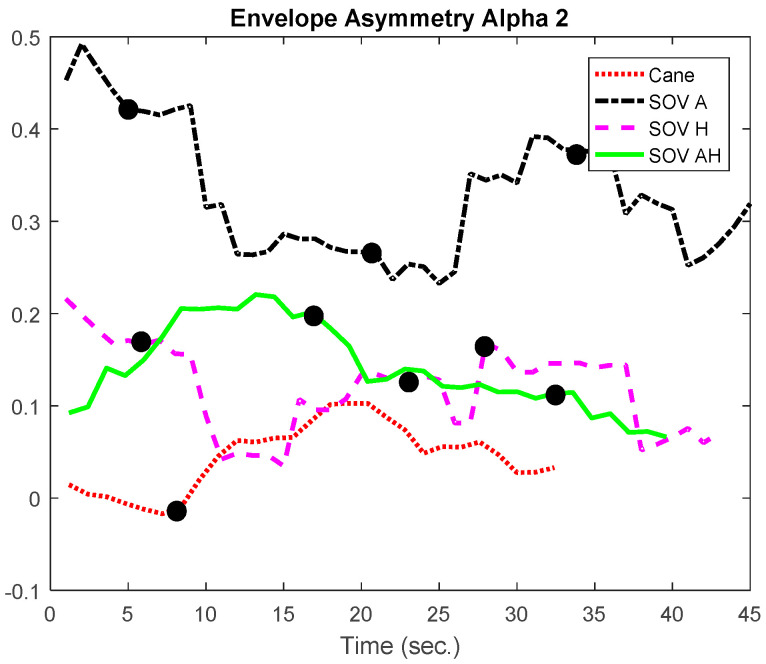
The envelopes of the alpha2 asymmetries for a user who was born blind.

In Figure 18 and Figure 19, the envelopes of the asymmetries depicted are related to a user who was born blind (early-blind). He usually navigates using the cane, and he took part in all the training sessions in the virtual environment and ego-static real-world tests. *A significant difference between navigation with the cane and the SoV device was observed only for the audio encoding, in terms of response inhibition and attentional demands*. Although SoV is a completely new device that implies a different way of navigation, however, the consistent training in the virtual environment and the ego-static real-world tests helped a lot the user to accommodate to the encodings. *The greater attentional demand (reflected by the alpha1 waves) was evident for audio stimulation,* and it could be assumed that this was due to the fact that the VIPs usually rely heavily on the environmental noise when they navigate. They also try to perceive natural noises when the SSD sonification is conveyed to them. From the perspective of *alpha2 frequencies, meaning speed and task difficulty, for this user, for all the encodings, the values obtained for navigation with the SoV device were significantly higher than those obtained in the case of using the white cane*. Anyhow, this conclusion was expected, considering the novelty of the SoV system for the users and the fact that the VIPs walked relying on the white cane in a natural style for a long time. The collisions (marked with dark dots) were well correlated with the inflection points of the envelopes’ variations.

**Figure 20:**
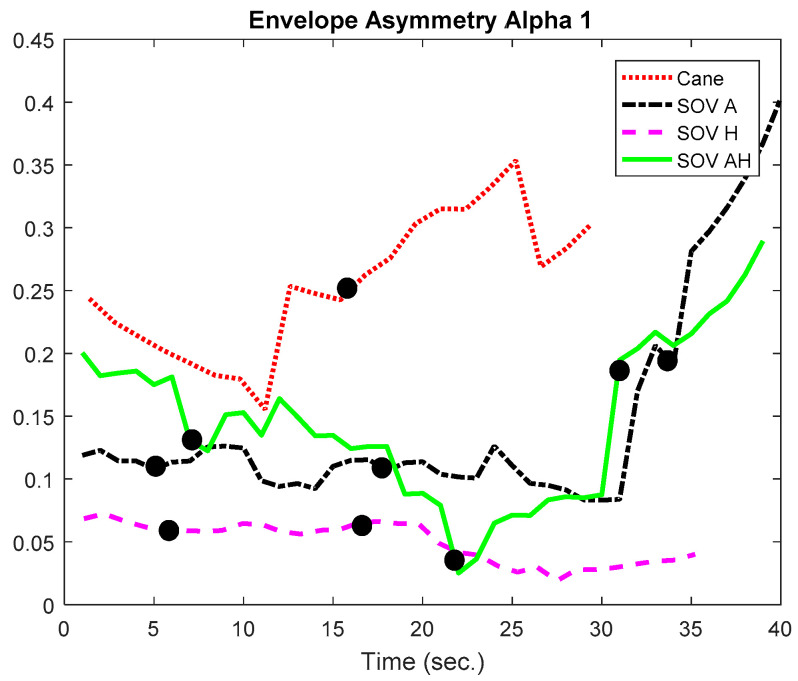
The envelopes of the alpha1 asymmetries for a late-blind user.

**Figure 21:**
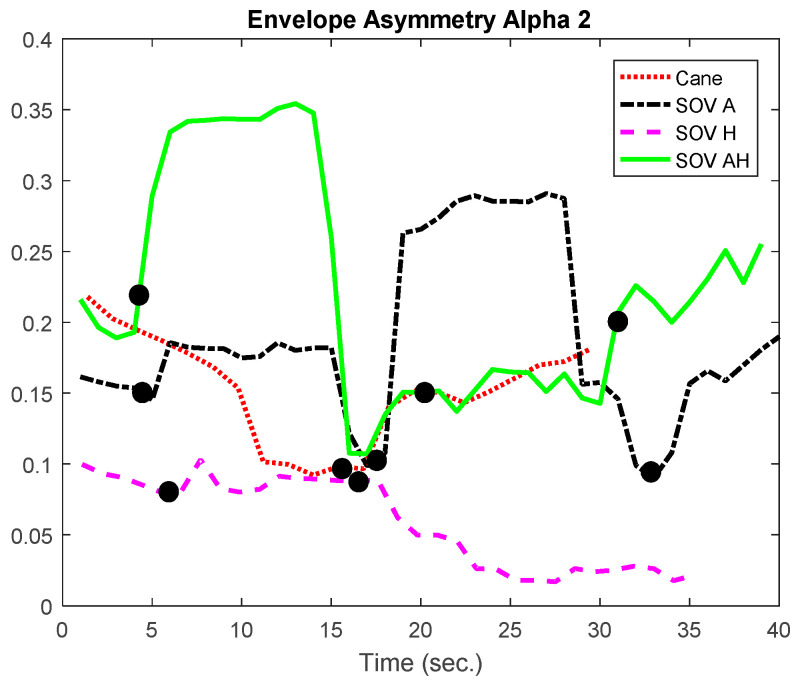
The envelopes of the alpha2 asymmetries for a late-blind user.

In Figure 20 and Figure 21, the envelopes presented correspond to a user from the late-blind group. He lost his sight at 17, has a good education, and usually navigates accompanied by a family member, without using the white cane. He quickly got used to the SoV device and got good scores in the training sessions. In this study case, the alpha1 asymmetry values (Figure 20) were higher for the cane navigation (even the necessary time was shorter), compared to those obtained for SoV navigation, regardless of how the environmental information was encoded. This demonstrated *a higher concentration for the cane navigation and good and fast accommodation with the SoV device*. *The alpha2 asymmetries (Figure 21) highlighted increasing difficulties for the audio and multimodal encoding tasks*. However, ***the range variations of the alpha1 and alpha 2 asymmetries were similar for the two users considered***. This suggested that ***an early and a late VIP had the same cognitive load, but there were differences between the navigation tasks: cane* vs. *SSD and between the different types of encodings (audio, haptic, and multimodal).***

**Figure 22:**
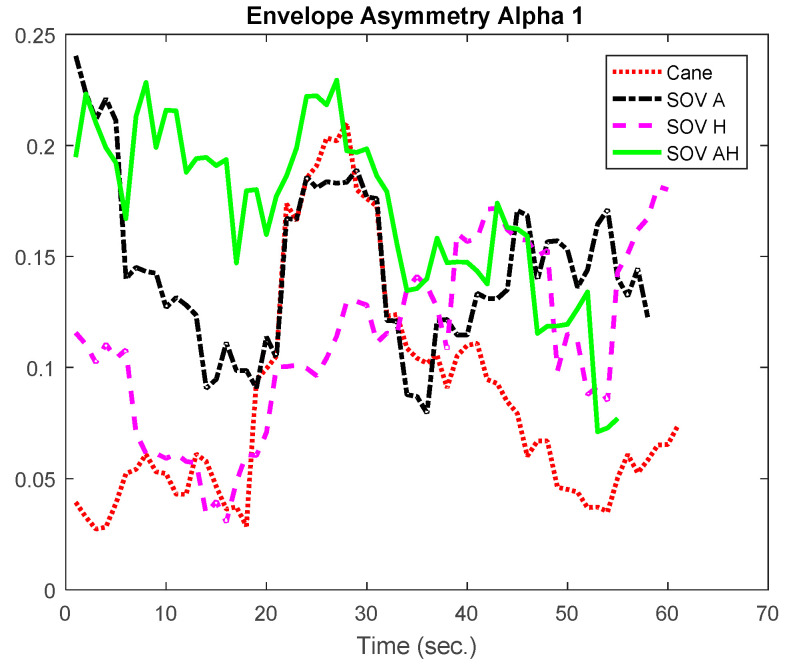
The average of the alpha1 asymmetry for all the users.

**Figure 23:**
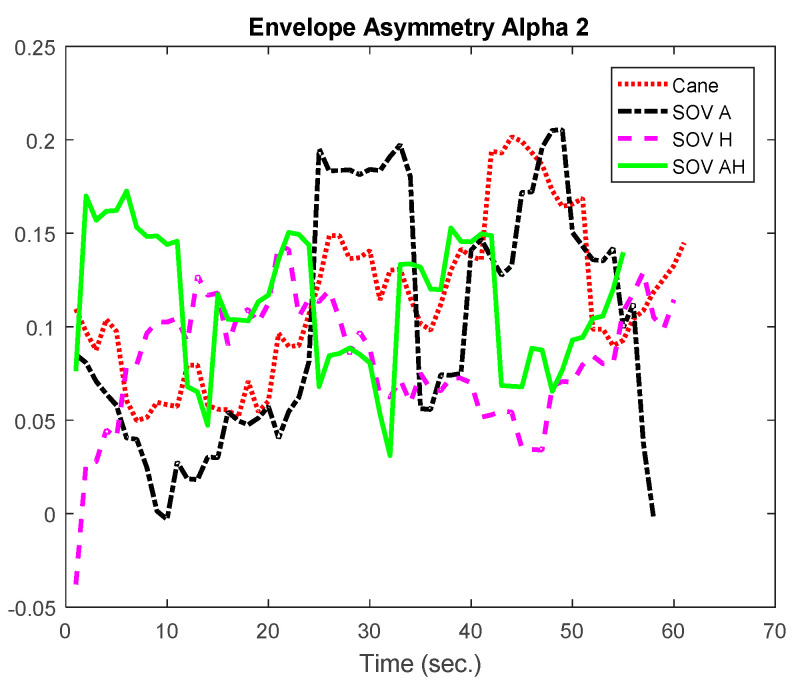
The average of the alpha2 asymmetry for all the users.

**Figure 24:**
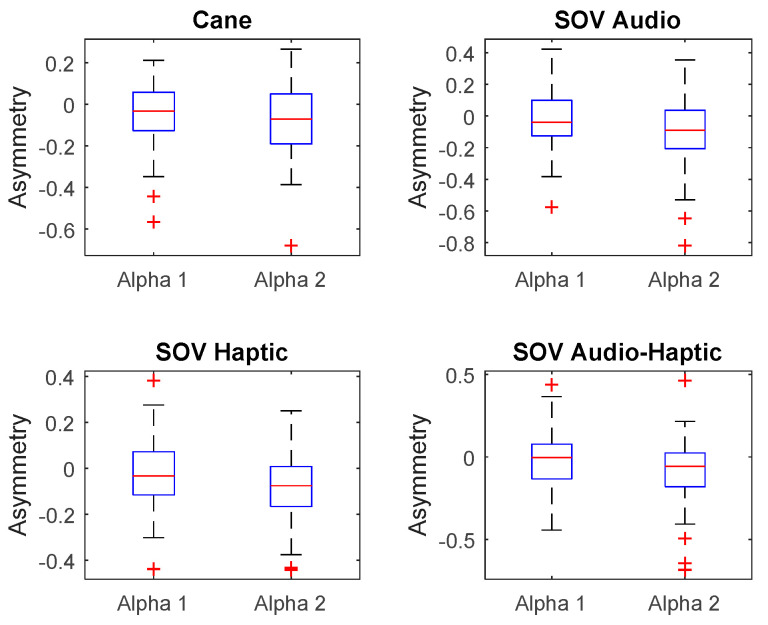
The envelopes for all the users, for scenario E.

**Figure 25:**
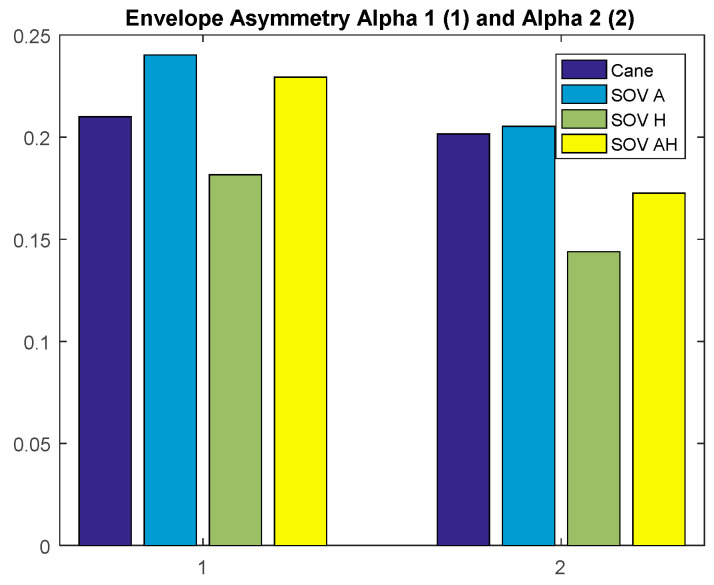
Average envelopes’ asymmetries for all users, for scenario E.

Besides the two particular cases presented above, an overview of the CL analysis is presented in Figure 22, Figure 23, Figure 24 and Figure 25. *By averaging the results for all the VIPs (early- and late-blind), in case of the most difficult trial (scenario E), it could be concluded that the alpha1 asymmetries for audio and multimodal codifications were a little higher (~0.2) compared to the cane and haptic modality (~0.1).* The variations within the whole asymmetries data set are displayed in the whisker plot from Figure 24.

### 6.4. Visual Cortex Activation Analysis

Some previous studies have revealed the presence of visual cortex activity in the case of the VIPs if a sensory substitution system creates an “information map” of the environment. Therefore, the total cognitive load (TCL) for electrodes O1 and O2 was investigated. Preliminary investigations of the experimental data showed that there was a major difference between the VC activity of the late-blind persons and of those who were born blind. Thus, a general conclusion regarding all VIPs could be drawn.

**Figure 26:**
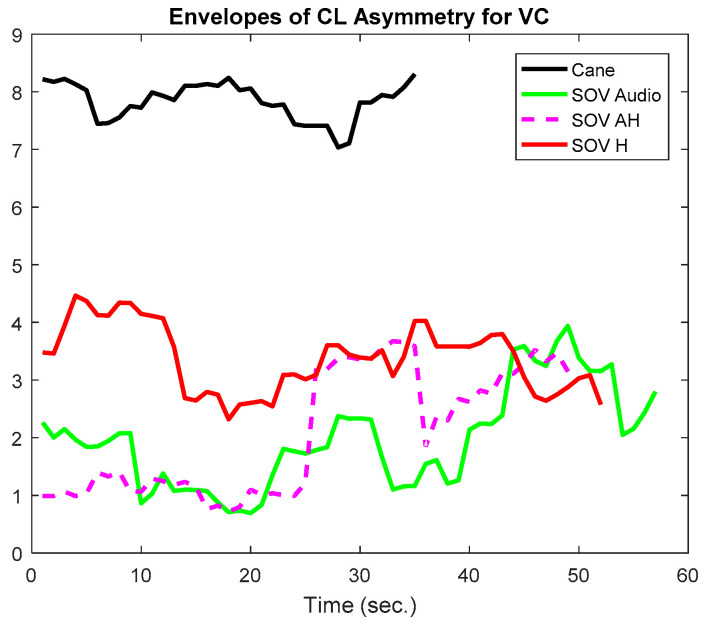
Visual cortex asymmetry for UserA (early-blind).

**Figure 27:**
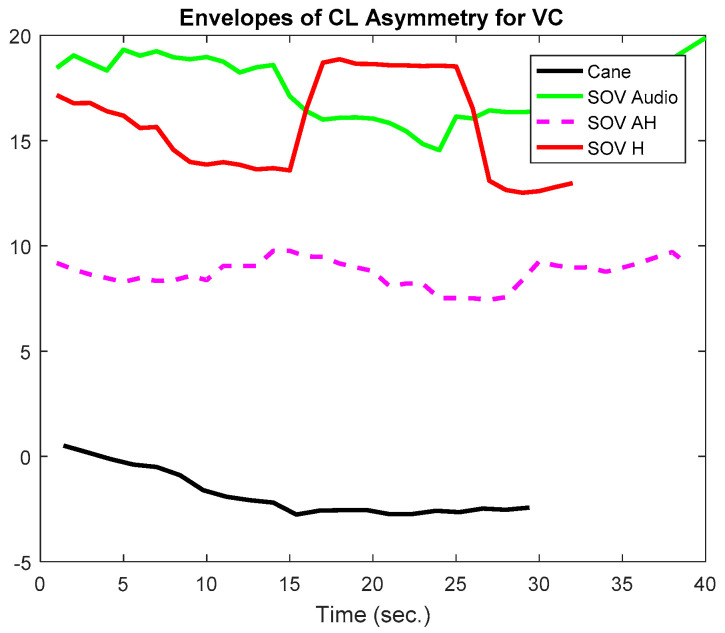
Visual cortex asymmetry for UserB (late-blind).

**Figure 28:**
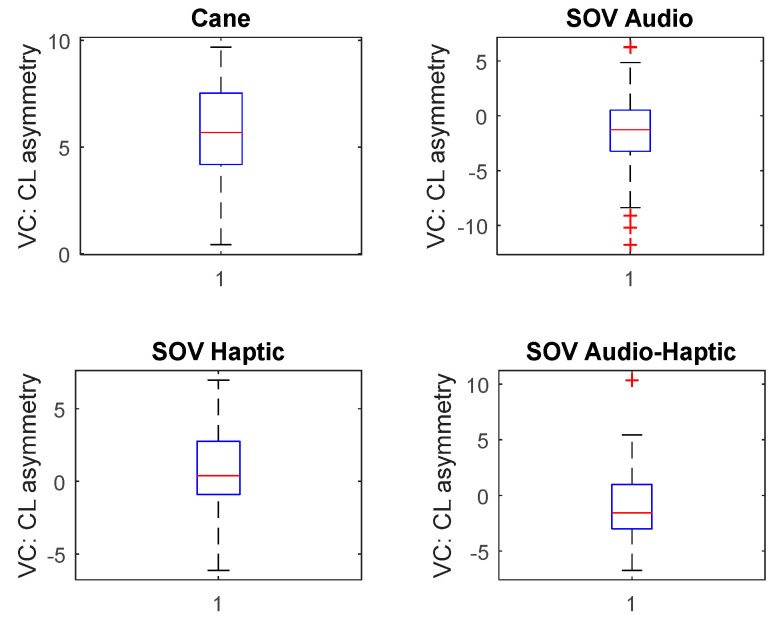
Visual cortex activity for UserA (early-blind).

**Figure 29:**
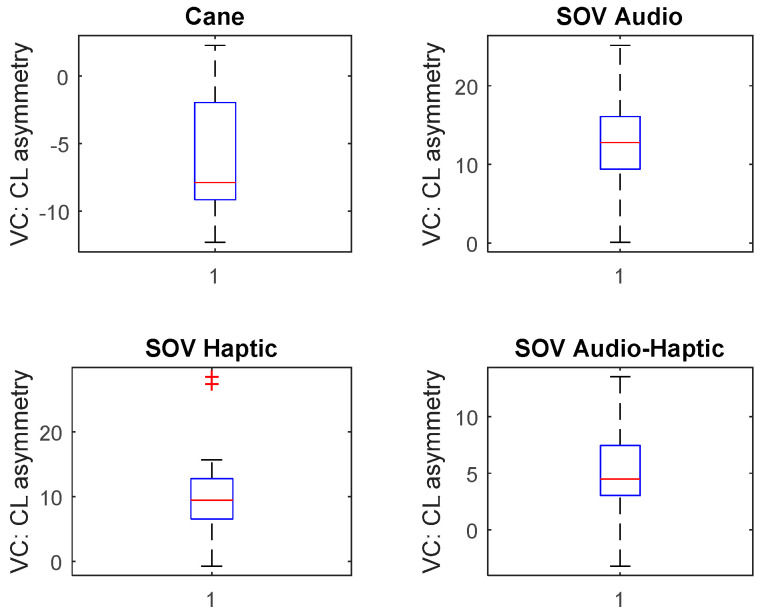
Visual cortex activity for UserB (late-blind).

The asymmetries of TCL in the visual cortex for UserA (early-blind) and UserB (late-blind) were calculated. In Figure 26 and Figure 27, the upper envelopes for TCL values are represented for UserA and UserB. *UserA was a born blind person, and, in his case, the asymmetry of the cane task was twice greater than the asymmetries of SoV tasks*. In line with some previous research, it is possible to associate the VC activity of the cane task with the fact that parts of his visual cortex were activated to refine his sensations and usual activities. In contrast, *the late-blind person’s VC activity in SoV tasks was much higher than in the cane task (which was negative) and more than five times higher than UserA’s visual activity*. The limited number of VIPs from each group (five early- and three late-blind users) did not permit to obtain valuable statistical results, but *for all the late-blind persons guided by audio and haptic stimuli, the average asymmetry of VC was around six times greater than that of the persons born blind*, as can be seen in Figure 28 and Figure 29. Another important observation was that *VC asymmetry was lower in the case of multimodal stimulation than in the case of separate stimulation (audio or haptic).* It must be emphasized that there was *no correlation between visual cortical activity and the number of collisions during navigation, regardless of the type of navigation or sensory input*.

### 6.5. Emotions Assessment During Real-World Navigation

As presented in the previous chapter, the emotional influence is a vast topic on which, from the dimensional perspective, the valence and arousal dimensions are advocated by Russell [66]. *Arousal* expresses calmness or excitement, whereas *valence* expresses a negative or positive effect. According to the comprehensive literature, *left frontal inactivation is an indicator of a withdrawal response*, which is often linked to a negative emotion, and *right frontal inactivation is a sign of an approach response* or positive emotion. Therefore, the **ratio of right and left asymmetry (valence state—VS**) was computed with the equation:

(2) $$VS=log\left(\frac{PS_{R}}{PS_{L}}\right)$$

where *PS_R_* and *PS_L_* are the power spectrum values of the right and the corresponding left hemisphere channels in a specific frequency band. The channels T7-T8, which are considered the most relevant for emotions assessment, but also C3, C4, P3, P4, F3, and F4, were considered based on the theoretical statements from the previous chapter [62].

In addition, the HR and EDA signals acquired during the tests were processed according to the standard approaches described in the literature [65]. The root mean square of the successive differences (RMSSD) values were calculated for the HR recorded using the Shimmer sensor. A low RMSSD value means a high HR, denoting a strong concentration, emotion, or physical effort, whereas a high RMSSD value corresponds to resting or to a relaxing activity. The HR values in the resting state for the involved users were different according to their age and personal rhythm. Therefore, the percentage of variation of RMSSD compared to the resting state was calculated. By pre-processing the signals acquired with Shimmer, the EDA signals (µS) were obtained. Then, the deconvolution performed using the Ledalab software provided the phasic and tonic components and skin conductance responses (SCRs)—abrupt increases in the conductance of the skin, measured in µS, were calculated.

**Figure 30:**
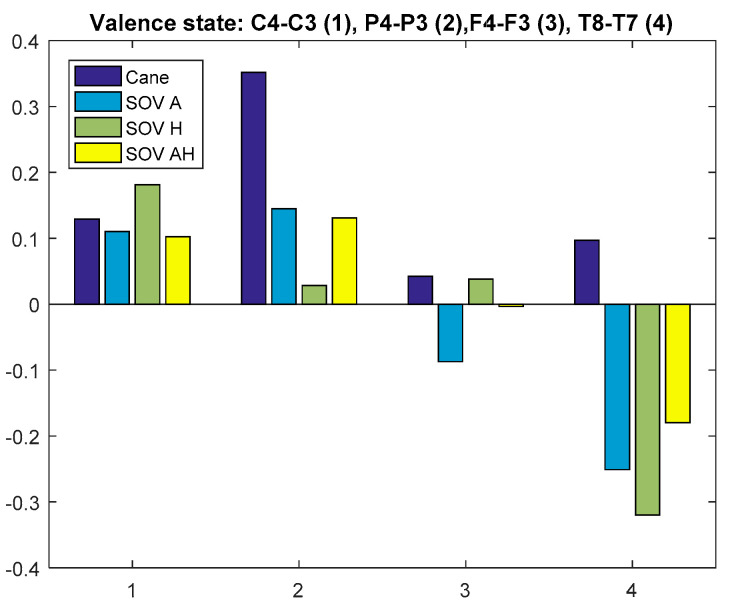
Average valence state (VS) for all visually impaired people (VIP).

Firstly, the global VS of all the users and all the performed tests was calculated for the chosen pairs of electrodes. The result is graphically depicted in Figure 30. The C3 and C4 electrodes were considered because their waves could be associated with hippocampus activity, together with T8-T7, which obviously are the most relevant for assessing emotions [65]. The VS calculated using the T8-T7 pair (T8 in the right hemisphere denotes negative emotions and lack of motivation, in contrast to T7) indicated a *low positive valence for cane navigation and a negative valence for SoV navigation*, in accordance with the cumulative time, distance, and the number of collisions from Table 1. For the *parietal lobe, the P4-P3 pair indicated a moderate VS for cane navigation and low VS for SoV navigation*. The pair C4-C3 reflected a low positive valance, close to the neutral state. The same observation applied for the F4-F4 pair, for which the low negative valence in the SoV navigation using the audio encoding must be taken into account.

The global percentage variation rate of RMSSD compared to the resting state decreased with: 21% for cane navigation, 39% for SoV navigation using the audio encoding, 44% for SoV navigation using the haptic encoding, and 41% for SoV navigation using the multimodal encoding. By computing the global SCR index, the following values were obtained: 0.18 for white cane navigation, 0.61 for SoV navigation using the audio encoding, 0.76 for SoV navigation using the haptic encoding, and 0.71 for SoV navigation using the multimodal encoding.

The trials were performed randomly within the same navigation type (cane or SoV) and for the same user, and no significant differences of the RMSSD values were remarked between the trials, even if they had different durations. Moreover, a slight increase (corresponding to an HR decrease) was observed towards the end of most of the tests. The users were not subjected to intense physical activity because they walked on the plain ground; however, the average of HR values was a little bit increased in comparison to the VTE tests. On the other hand, the EDA signals were sensitive to most of the collisions, especially in the case of the SoV navigation.

**Figure 31:**
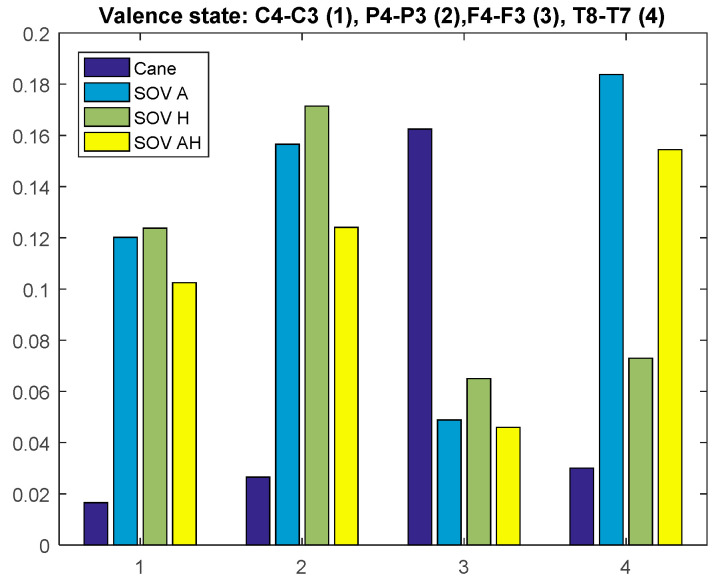
Average VS of UserA.

**Figure 32:**
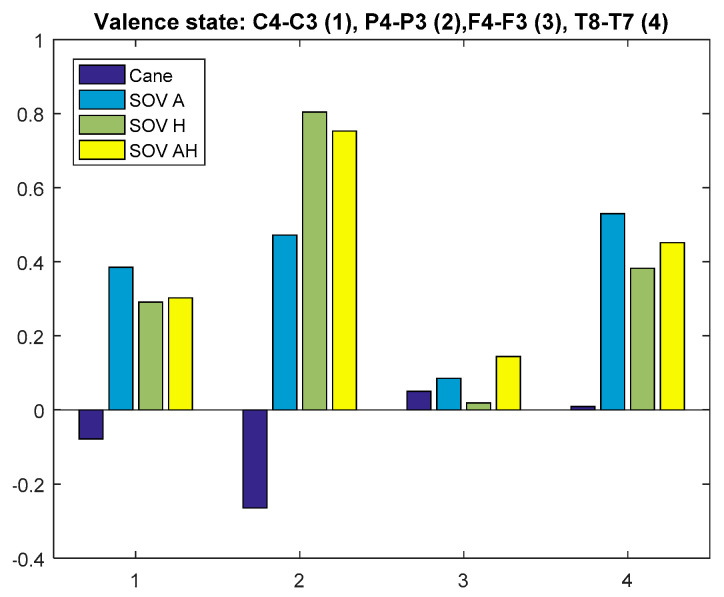
Average VS of UserB.

In Figure 31 and Figure 32, we present the VS values for the two special users (UserA and UserB). In the case of UserA, who was intensively trained in the VTE and in the RW, the *valence was positive but very close to the neutral state for all the navigation types, with the remark that the valence for SoV navigation was a little bit higher than for cane navigation*. His RMSSD and SCR values did not differ significantly between the navigation modes.

Analyzing the results for UserB, who is usually guided by a family person, it is obvious that for him, navigating using the white cane induced a neutral to low negative valence, and navigating using the SoV device gave him more security, satisfaction, and a more comfortable state. This was underlined by a decrease of the RMSSD percentages and an increase of SCRs for all navigation modes.

**Figure 33:**
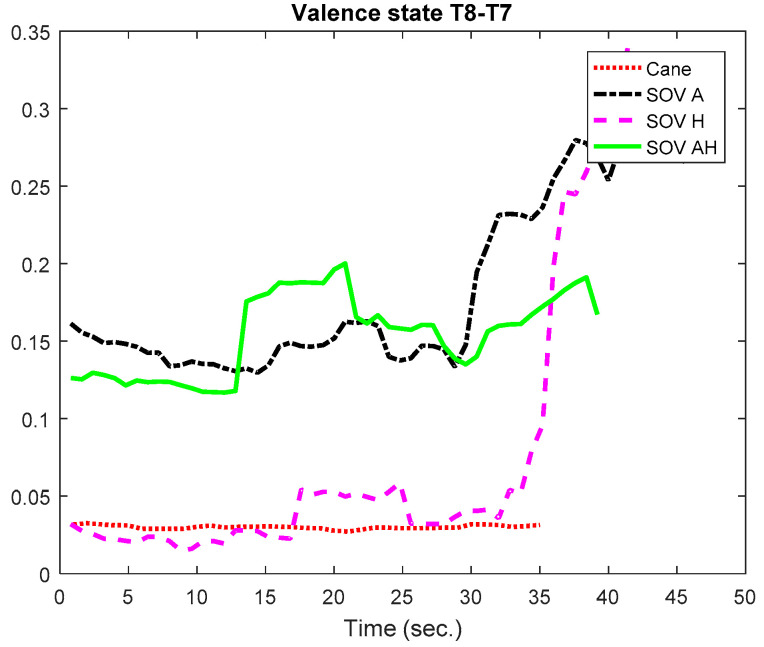
VS evolution in time for UserA.

**Figure 34:**
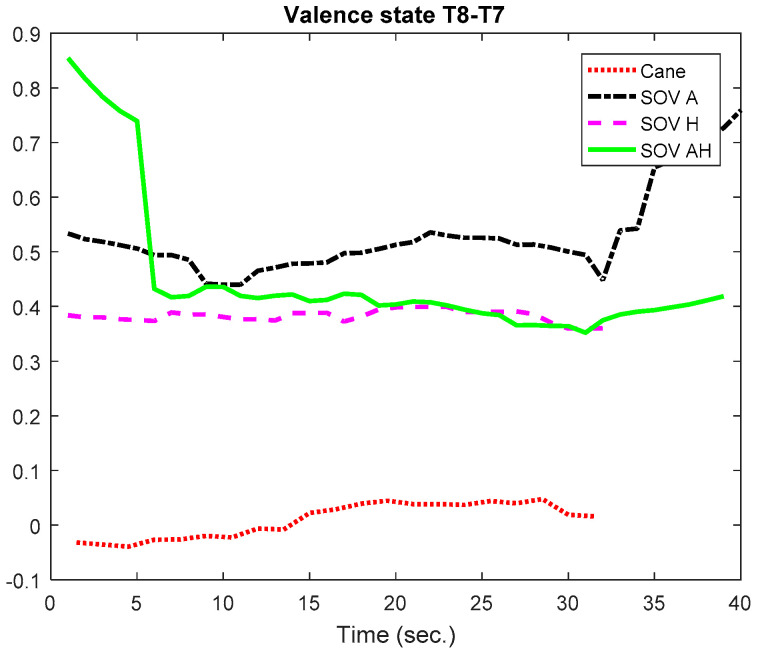
VS evolution in time for UserB.

In Figure 33 and Figure 34, the evolution in time of the VS for the T8-T7 pair is represented for UserA and UserB in order to highlight the slow evolution of valence during a trial. In general, the collisions did not essentially affect the valence changes, as in the case of the cognitive load assessment.

### 6.6. Limitation of This Study

The limitations of the current study reside in the fact that we performed the tests with a small number of users for both categories (early-blind and late-blind). Although the results were interesting and in line with the existing literature, a more thorough evaluation should be realized. In addition, the EEG recordings were performed using a limited number of electrodes. As future directions, we plan to use a more advanced EEG recording device, with a higher number of electrodes, and to improve the SoV device so that it would be lighter and more comfortable to be worn.

The results of real-world experiments were strongly influenced by a consistent training period, similar to all users, which requires a great deal of time. Future work can extend the realistic scenarios of RW traveling for enhancing the impact of the study.

## 7. Conclusions

This paper presented an experimental framework and a study based on EEG, HR, and GSR signal analysis, aiming to assess the brain cortex activation and affective reactions of the visually impaired persons to the stimuli provided by a sensory substitution device used for navigation in real-world scenarios, compared to the white cane navigation. The study was focused on the evaluation of working memory load, visual cortex activation, and emotional experience when the VIPs perceived audio, haptic, and multimodal stimuli during a navigation task in five different types of scenarios.

The choice of the Brain-Computer Interface (BCI) equipment proved to be inspired because its characteristics allowed a good acquisition of EEG signals simultaneously with the use of the SoV device. The same BCI equipment has been employed successfully in other studies of our own concerning multimodal neuromotor rehabilitation [67,68]. An important feature of the experimental setup is the ability to synchronize the data streams and to align the acquired signals with the events extracted from the video recordings. The training performed in the VTE and the ego-static tests performed indoors had an essential role in preparing the users to perceive distances, positions, and object dimensions only by means of the audio, haptic, and multimodal stimuli, giving confidence to all of them in using the SoV device. The aim was to provide all users the ability to automatically understand the complexity of a scene. Besides, during the VTE training, multiple resting sessions were recorded for all volunteers, which had an important role in establishing a baseline.

The perception of audio and haptic stimuli using the SoV device was assessed in terms of cognitive load, pleasantness, excitement, and events, for all the visually impaired users, as well as for the specific categories (early-blind or late-blind). *All in all, the haptic stimuli appeared to be less intuitive than the audio stimuli.*

The analysis showed that *navigating with the SoV device increased the cognitive load and the working memory (lower accuracy and longer response times).* The analysis of the EEG data revealed *the usage of verbal working memory in the posterior parietal cortices*. The obtained results indicated that the left-right asymmetry of the prefrontal cortex had distinguishable characteristics when the VIPs were navigating in real-world *environments with a wide range of obstacles*.

The visual cortex exploration revealed *a significant activation when using the SoV device, only for the late VIPs*. The low VC activity of congenitally blind persons during SoV navigation could be related to brain plasticity, which allows the auditory and somatosensory systems to extend their functionality in that part of the cortex.

Finally, we assessed the valence state of the users when navigating in unfamiliar indoor environments based on mobile monitoring and a fusion of EEG and physiological (EDA and HR) signals. *For the generic VIP population, the use of the SoV device induced a low negative valence in contrast with cane usage*. But the findings differed for the specific categories of sight loss (early- and late-blind), pointing out the particular needs/difficulties faced by each category of VIP.

This study proved once more that sensory substitution is an alternative method, which helps the blind people to acquire information about the surrounding space and to navigate independently in unknown real-world environments, safely and comfortably, after substantial training.

The findings hopefully empower the knowledge of how the visually impaired persons are stressed and emotionally affected by SSD navigation and contribute to the development of the intelligent navigation devices, aiming for the VIPs’ safety and well-being. The results of our work can inspire researchers working in the field of IoT devices comprising sensors, antennas, and Bluetooth, which have created navigation rules based on a fuzzy controller [69], GPS embedded in a stick with voice recognition for obstacles detection [70], computer vision-based assistants [71], or assistive systems relying on wearable smart glasses and mobile applications [72].

Valuable research projects have investigated the efficiency of intelligent sensory substitution devices [73], and, in this context, our research brought an important contribution by analyzing EEG and physiological signals in order to assess the cognitive effort and emotional state of users in real-world navigation.

## Figures and Tables

**Table 1:** Cumulative experimental data for the treasure hunt (TH) tasks—navigation with the Sound of Vision (SoV) device and white cane.

Codification	Scenario Type	Collisions Total Number	Path Total Distance (m)	Total Time (s)
Audio	A	12	33.69	293
	B	10	44.36	299
	C	22	42.8	261
	D	18	41.8	306
	E	20	62.05	409
Haptic	A	13	28.3	179
	B	11	39.1	221
	C	17	41	261
	D	20	44.1	255
	E	34	54	440
Audio and Haptic	A	7	33.35	236
	B	14	39	267
	C	15	47.3	327
	D	12	51.6	271
	E	24	56.5	345
White cane	A	4	23.27	204
	B	5	28.8	205
	C	6	27.8	213
	D	2	30.16	206
	E	6	37.77	255
